# Emerging Technologies for Genome-Wide Profiling of DNA Breakage

**DOI:** 10.3389/fgene.2020.610386

**Published:** 2021-01-27

**Authors:** Matthew J. Rybin, Melina Ramic, Natalie R. Ricciardi, Philipp Kapranov, Claes Wahlestedt, Zane Zeier

**Affiliations:** ^1^Department of Psychiatry and Behavioral Sciences, University of Miami Miller School of Medicine, Miami, FL, United States; ^2^Center for Therapeutic Innovation, University of Miami Miller School of Medicine, Miami, FL, United States; ^3^Institute of Genomics, School of Biomedical Sciences, Huaqiao University, Xiamen, China

**Keywords:** genome instability, DNA damage, double strand break (DSB), single strand break (SSB), neurodegeneration, neurodegenerative disease, aging

## Abstract

Genome instability is associated with myriad human diseases and is a well-known feature of both cancer and neurodegenerative disease. Until recently, the ability to assess DNA damage—the principal driver of genome instability—was limited to relatively imprecise methods or restricted to studying predefined genomic regions. Recently, new techniques for detecting DNA double strand breaks (DSBs) and single strand breaks (SSBs) with next-generation sequencing on a genome-wide scale with single nucleotide resolution have emerged. With these new tools, efforts are underway to define the “breakome” in normal aging and disease. Here, we compare the relative strengths and weaknesses of these technologies and their potential application to studying neurodegenerative diseases.

## The DNA Damage Response

Aberrant modification of DNA is a natural and common consequence of many cellular processes such as cellular respiration and transcription. Left unchecked, DNA damage can introduce mutations or compromise overall genome stability (Aguilera and García-Muse, 2013). To rapidly respond and mitigate the negative consequences of DNA damage, a sophisticated array of signaling pathways have evolved that are aptly referred to as the DNA damage response (DDR) (Jackson and Bartek, 2009). Disruption of DDR signaling, or subsequent repair processes, are linked to cancers and neurological disorders including ataxia telangiectasia (AT), amyotrophic lateral sclerosis (ALS) and Alzheimer's disease (AD) [see (Terabayashi and Hanada, 2018) for review of genome instability syndromes]. In some cases, mutations in the same DDR-related gene are associated with both cancer and neurodegeneration. For example, mutations in the gene encoding ATM (ataxia telangiectasia mutated) can lead to the development of AT or breast cancer (Renwick et al., 2006; Choi et al., 2016), while mutations in the gene encoding FUS (fused in sarcoma) cause ALS and sarcomas (Ward et al., 2014). The reasons why cancer and neurodegenerative disease are linked are not fully-understood, but genome instability increases with age and is a major risk factor for both forms of disease (Hanahan and Weinberg, 2011; Niedernhofer et al., 2018). Neurons are particularly susceptible to incurring DNA damage as a result of high metabolic and transcriptional output and their incompatibility with homologous recombination (HR), a replication-dependent, high-fidelity DNA repair pathway (Madabhushi et al., 2014; Maynard et al., 2015). A major limitation that has prevented a greater understanding of DNA damage and repair in disease states has been the inability to map different forms of DNA breaks on a genome-wide scale. In recent years, however, advancements in techniques for mapping DNA breakage with unprecedented scope and accuracy have opened new avenues for investigation that will increase our understanding of DNA breakage and repair. Here, we review these genome-wide DNA damage detection technologies and their current and future applications with an emphasis on neurodegenerative disease.

## Causes and Consequences of DNA Damage

The term DNA damage encompasses various alterations of nucleic acids including single strand breaks (SSBs), double strand breaks (DSBs) and a host of aberrant chemical modifications that can cause DNA breaks or are cleaved during repair (thus mimicking DNA breaks). The most deleterious forms of DNA damage, and focus of this review, are SSBs and DSBs [for comprehensive review of types DNA damage and repair pathways see (Chatterjee and Walker, 2016)]. The relative contribution of SSBs vs. DSBs is disease specific, but, in general, SSBs occur more frequently and are less genotoxic than DSBs. To mitigate the effects of DNA breakage, multiple repair pathways with various degrees of efficiency and fidelity have evolved (Tiwari and Wilson, 2019). When these pathways are overwhelmed or ineffectual, accumulating DNA damage can lead to mutation and may trigger apoptosis. The DDR intersects with apoptotic signaling pathways in various ways. Generally, DDR-mediated apoptosis consists of DNA damage detection through sensor proteins that subsequently activate mediator and effector proteins. Mediator and effector proteins then facilitate repair through transcriptional activation or mobilization of repair proteins. Importantly, mediator and effector proteins can also induce the expression of pro-apoptotic genes. As with most cellular signaling pathways, negative feedback loops prevent over-activation of the DDR for routine damage, however, if damage persists or continues to accumulate, apoptosis may ensue. For example, DNA damage activates ATM and subsequently, the tumor protein p53. Pervasive activity of p53, in turn, activates various genes including DNA damage repair proteins, pro-apoptotic factors and MDM2 (a p53 inhibitor). The MDM2 negative feedback loop prevents apoptosis from being triggered by routine DNA damage but not for excess damage. For a review of this and other DDR-mediated apoptosis pathways see (De Zio et al., 2013). In addition to apoptosis, accumulating or irreparable DNA damage may also lead to cell senescence (Surova and Zhivotovsky, 2013) a phenotype that has been observed in DNA damage-laden post-mitotic neurons (Jurk et al., 2012; Fielder et al., 2017). However, the molecular determinants of senescence or apoptosis in response to DNA damage are not fully characterized. Overall, given that a cell is consistently exposed to exogenous and endogenous sources of DNA damage, an effective DDR is crucial for maintaining cell viability.

The link between DNA damage and neurodegeneration arises, in part, from the unique pressure that neurons are under to maintain genomic stability. High oxygen consumption, pervasive transcription and longevity make neurons intrinsically predisposed to accumulating DNA damage (Rass et al., 2007; Ciccia and Elledge, 2010). Moreover, as a consequence of their inability to utilize high-fidelity homologous recombination (HR) repair, whereby the sister chromatid serves as a template during cell division, post-mitotic neurons are assumed to utilize the efficient but mutagenic non-homologous end joining (NHEJ) repair pathway to near exclusion. Neurons are nonetheless capable of utilizing other repair pathways including single strand annealing (SSA) and microhomology-mediated end joining (MMEJ) that require small stretches of sequence homology (<25 nucleotides) to direct repair (Bhargava et al., 2016). Homology-directed DNA DSB repair pathways appear to be particularly relevant for the repair of transcribed DNA and may play a role in neurodegenerative disease (Keskin et al., 2014; McDevitt et al., 2018; Welty et al., 2018; Yasuhara et al., 2018; Andrade et al., 2020). Overall, accumulating DNA damage is characteristic of many neurological disorders, including aging and age-related neurodegenerative disease [for review consider (McKinnon, 2017)]. However, our mechanistic understanding of the repair pathways utilized by neurons, the determinants of repair pathway choice and perturbations to these pathways in disease remain obscure.

The outcome of DDR signaling is cell-type and context-dependent with many ill-defined characteristics. This, as well as the strong connection between dysregulation of the DDR and neurodegeneration, warrants continued research to better understand how aberrant neuronal DDR signaling and DNA damage repair contribute to disease. Until recently, a major barrier to such research has been the inability to profile DNA damage genome-wide at single nucleotide resolution. To facilitate these efforts, several new techniques have been developed to define the cellular “breakome”—or genome-wide DNA damage profile. These new technologies have provided unprecedented scope and resolution that will increase understanding of how DNA damage contributes to disease, including neurodegenerative diseases. Here we review these techniques and discuss their potential implications for future research efforts.

## Indirect DNA Damage Profiling Methods

Previously, detecting the location of DNA damage genome-wide largely relied on ChIP-seq (chromatin immunoprecipitation and sequencing) of DNA fragments associated with DNA repair machineries, such as p53-binding protein 1 or the phosphorylated variant histone H2AX (γH2AX). While able to detect DSBs genome-wide, this method is indirect and unable to identify lesions at single-nucleotide resolution. Other indirect DNA damage profiling techniques include translocation-capture sequencing (TC-Seq) (Klein et al., 2011), GUIDE-seq (Tsai et al., 2015), integrase-defective lentiviral vector (IDLV)-mediated DNA break capture (Wang et al., 2015) and linear amplification-mediated high-throughput, genome-wide, translocation sequencing (LAM-HTGTS) (Frock et al., 2015; Hu et al., 2016), all of which detect DSBs or chromosomal rearrangements and translocations by analyzing the products of non-homologous end joining repair (NHEJ). While NHEJ is a major repair pathway utilized by mature, post-mitotic neurons, these methods may miss lesions repaired through other pathways. Nonetheless, these methods can still be applicable for understanding mechanisms of DNA damage in relation to neural development and degeneration. For example, a recent study by Tena et al. incorporated the LAM-HTGTS method to detect recurrent genome break clusters, or RDCs, in primary mouse neural stem and progenitor cells (Tena et al., 2020). The LAM-HTGTS method detects genome-wide “prey” DSBs via their translocation to a fixed Cas9/single-guide RNA-generated “bait” DSB. The bait-prey complex is cloned directly from isolated genomic DNA using LAM-PCR and unidirectionally ligated to bridge adapters in preparation for Illumina Miseq paired-end sequencing. A bioinformatics pipeline then identifies “prey” sequences that contribute to the prey-bait complex and maps them across the genome. Using this method, Tena and colleagues identified 29 RDCs and their locations in neural progenitor cells deficient in NHEJ repair protein XRCC4 (X-ray repair cross-complementing protein 4) and p53, further characterizing the unknown mechanism of neural RSC-gene breakage and its relation to neurological diseases and brain cancer. Indirect and NHEJ-focused DNA damage profiling techniques may, therefore, remain applicable to specific scientific questions pertaining to the NHEJ repair pathway in mature neurons.

## Low Resolution DSB Detection

Low resolution techniques for detecting DSBs genome-wide include dDIP (damage DNA immunoprecipitation; 2011) (Leduc et al., 2011), DSB-seq (2014) (Baranello et al., 2014), Break-seq (2015) (Hoffman et al., 2015), dBrIC (DNA break immunocapture; 2016) (Grégoire et al., 2016) and BrITL (breaks identified by TdT labeling, 2018) (Shastri et al., 2018). While sample preparation or methods of analysis vary between individual methods, each of these techniques involves labeling DSBs with biotinylated nucleotides using the terminal deoxynucleotidyl transferase (TdT) DNA polymerase. The labeled DNA is then fragmentated (via sonication or enzymatic digestion), and labeled fragments are immunoprecipitated using an anti-biotin antibody. Captured fragments are prepared for sequencing and DSBs can then be detected with an average resolution of ~100–300 base pairs (bp), depending on the method. Many groups have utilized these techniques to study DNA damage throughout the genome. Hoffman and colleagues utilized Break-seq to reveal genome instability caused by conflicts caused by simultaneous replication and transcription (Hoffman et al., 2015). After adapting Break-seq to mammalian cells, Chakraborty et al. used Break-seq to investigate global chromosome breakage in fragile X syndrome patient-derived cells, discussed in more detail below (Chakraborty et al., 2020). Shastri et al. used BrITL to identify genomic regions prone to DNA breakage during replication by determining where DSBs accumulate after inhibition of the ATR checkpoint kinase which sustains DNA structures and prevents damage when replication is stalled. They found that repetitive DNA relied most on ATR to avoid breaks—potentially implicating ATR in the many nervous system diseases that are associated with expanded microsatellite repeats, such as Huntington's disease, fragile X syndrome, frontotemporal dementia and amyotrophic lateral sclerosis. On the whole, these techniques showcase the rapid advancement of DSB mapping techniques and their improved resolution compared to indirect methods.

## Nucleotide Resolution DSB Detection

### BLESS

**Figure 1:**
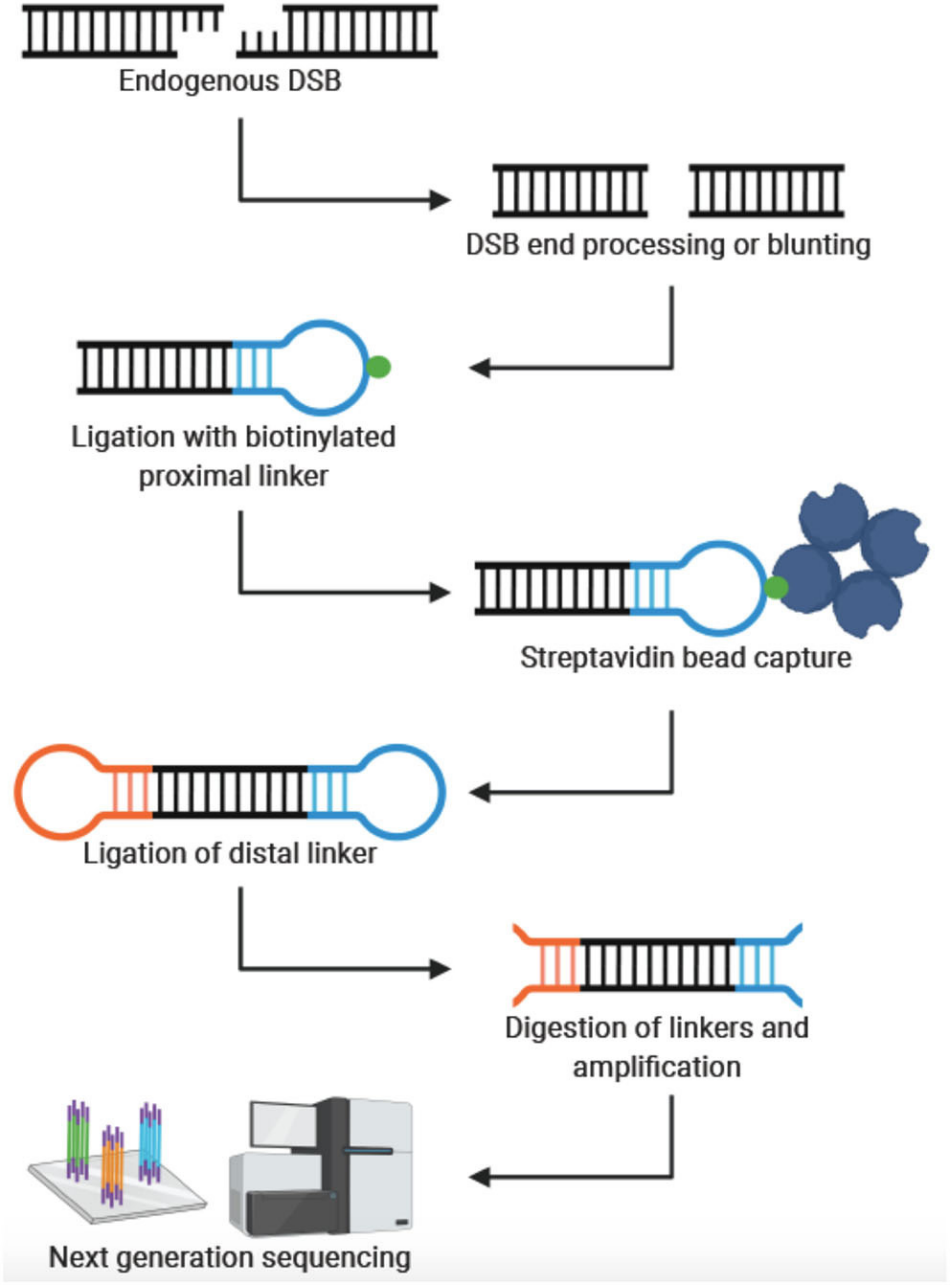
General workflow for genome-wide, nucleotide-resolution detection of DSBs. Created with BioRender.com.

In 2013, Crosetto et al. were the first to develop a genome-wide, nucleotide resolution DSB mapping technique they called BLESS (direct *in situ* breaks labeling, enrichment on streptavidin and next-generation sequencing) (Crosetto et al., 2013). Using BLESS, a unique “breakome”, or genome-wide DNA damage profile can be generated. In this method, cells are fixed to stabilize the chromatin and prevent artificial DSBs. After fixation, nuclei are isolated, and DSBs are blunted and 5′-phosphorylated. A biotinylated linker is then ligated to the blunted DSB ends using the T4 ligase enzyme—an enzyme specific for ligating double-stranded but not single-stranded DNA. The linker consists of a barcode sequence followed by a recognition site for the endonuclease *XhoI*. Genomic DNA is then extracted, and the biotinylated fragments are captured with streptavidin beads. A second distal linker containing a *XhoI* cut site is ligated to the free end of the captured fragment. After the linkers are digested with *Xhol*, fragments are prepared for Illumina sequencing by PCR. Crosetto et al. validated their protocol in HeLa cells and mouse B-lymphocytes. B-lymphocyte activation induces DSB in the immunoglobulin heavy chain (IgH) donor Su region and the downstream acceptor S region. As expected, DSBs were significantly enriched in those regions compared to the average DSB density throughout the genome. The development of BLESS was an enormous breakthrough—allowing DSBs to be mapped genome-wide at nucleotide resolution in human cells for the first time. The significance of BLESS is evidenced by the fact that all subsequent genome-wide DSB detection methods share common features with BLESS (Figure 1).

### End-seq and DSBCapture

In 2016, Canela et al. developed END-seq—which reduces the necessary input DNA by 2-fold, avoids fixation through use of agarose plugs and streamlines the adaptor ligation step. Together, END-seq increased the sensitivity of DSB detection as indicated by a 36-fold increase in the average proportion of total reads mapping to AsiSI restriction sites in a direct comparison experiment with BLESS. AsiSI endonuclease restriction is a common validation method used for genome-wide break mapping studies because it is a highly specific endonuclease with 1,222 predicted cleavage sites in the human genome. The authors also demonstrate that attaching A-tails to DSBs preserves the ends of the DNA breaks more faithfully than BLESS. However, END-seq still requires overhang blunting prior to adaptor ligation, making asymmetrical end structure identification less precise. While the method can detect low-level genome-wide DSBs, the authors conclude that END-seq is most sensitive when interrogating site-specific breaks like recombination-activating gene (RAG)-induced DSBs necessary for V(D)J recombination at antigen receptor genes and even the lower frequency off-target RAG-induced breaks. Their studies on RAG-induced DSBs also showed that END-seq can differentiate between RAG binding and RAG activity. They also overlaid AsiSI-mediated and RAG-mediated DSB sites with multiple histone marks to show that endonuclease mediated DSBs depend significantly on chromatin accessibility in addition to target sequence. END-seq is semi-quantitative and capable of detecting 1 DSB per 10,000 cells which is sufficiently sensitive in CRSPR-Cas9 studies to detect off-target cutting, end resection dynamics, and genotoxic drug mechanisms. Around the same time as END-seq, Lensing et al. published a similar technique, termed DSBCapture, that also featured A-tailing of DSBs, although without the use of agarose plugs, to increase the sensitivity and reproducibility of DSB mapping compared to BLESS (Lensing et al., 2016). Together, END-seq and DSBCapture represent valuable improvements for genome-wide DSB mapping.

### BLISS

Not long after END-seq and DSBCapture, Yan et al. introduced a technique called BLISS (breaks labeling *in situ* and sequencing) (Yan et al., 2017). In contrast to previous methods, BLISS begins with fixed cells or tissue sections attached to microscope slides or coverslips. Because of this, BLISS does not require cell lysis through centrifugation, thereby minimizing the risk of forming artificial DNA breaks. With this protocol modification, Yan and colleagues significantly reduced the amount of starting material needed, addressing a major limitation of previous methods which required a considerable number of cells, typically millions. Indeed, with BLISS, the authors located and quantified DSB ends generated by SpCas9 (*Streptococcus pyogenes* Cas9) in a sample of a few thousand transfected HEK293 cells. They also used BLISS to detect DSBs in U2OS cells treated with the topoisomerase inhibitor, etoposide. Notably, etoposide treatment caused DSBs to accumulate around transcriptional start sites (TSS); results which are consistent with previous findings (Yang et al., 2015). Another weakness of previous techniques is limited scalability. Yan et al. addressed this limitation by adapting the BLISS protocol to perform all *in situ* reactions in multi-well plates, thereby simplifying the workflow.

After fixing cells to slides or coverslips, subsequent steps in the BLISS method proceed similarly to previous protocols with DSB end blunting, adaptor ligation, gDNA purification and sequencing. Importantly, the BLISS adaptor contains three unique features: (1) a T7 promoter sequence for T7-mediated *in vitro* transcription in preparation for sequencing, (2) unique molecular identifiers (UMIs) which provides a semi-quantitative view of DSB frequency, and (3) a barcode that allows for sample multiplexing. The unique BLISS adaptor was an important advancement toward quantifying precise numbers of DSBs per cell rather than relative frequencies. However, the UMIs can vary from sample to sample and often lead to complex and costly sequencing and mathematical modeling. The work of Yan et al. appreciably improved DSB mapping by reducing the required input material and increasing scalability and precision of DSB quantification.

### i-BLESS and qDSB-Seq

In 2018, the BLISS technique was modified slightly to be more versatile by accommodating cells of varying sizes. The new method, i-BLESS (immobilized-BLESS) (Biernacka et al., 2018) can be utilized in various settings. For example, yeast nuclei are incompatible with the high-speed centrifugations required in BLESS, which would result in chromatin shearing within the nuclei. The i-BLESS method incorporates encapsulating cells in agarose, protecting DNA from mechanical damage. Additionally, this change makes the i-BLESS method more applicable for certain neuronal nuclei that are particularly small, such as granule cells.

In addition to i-BLESS, the same group recently introduced another technique called quantitative DSB sequencing (qDSB-Seq) designed specifically to quantify the exact frequencies of DSBs per cell. Until this time, quantification of precise DSB frequencies was still a costly and complex process (see BLISS). To solve this, Zhu et al. developed the qDSB-Seq protocol wherein site-specific DSBs (spike-ins) are induced with a known restriction enzyme. The cutting efficiency of the restriction enzyme and the frequency of spike-in DSBs is quantified with gDNA sequencing or qPCR. Then, both endogenous DSBs and the spike-ins are labeled and sequenced using any DSB detection method (e.g., BLESS or i-BLESS). In this way, the precise frequency of non-spike-in DSBs per cell is calculated using the pre-determined artificial spike-in frequency. To compare quantifications between the qDSB-Seq and BLISS methods, the authors induced DSBs in DIvA (AsiSI-ER-U2OS) cells by activating the restriction enzyme AsiSI by treating with 4-hydroxytamoxifen (4OHT). They found that qDSB-Seq quantification of DSB frequency per cell yielded comparable results to immunofluorescent assessment of γH2AX foci. In contrast, quantification using the BLISS method, estimated DSB frequency was three orders of magnitude less. Overall, both the i-BLESS and qDSB-Seq methods significantly improved the state-of-the-art of DSB mapping by expanding DSB mapping to any sized cell and providing a means of precisely quantifying the number of DSBs in individual cells.

### CNCC-seq

In January of 2020, Szlachta et al. presented the first technique for mapping endogenous DSB end structures at single nucleotide resolution (Szlachta et al., 2020). This method, known as coverage-normalized cross correlation sequencing (CNCC-seq), is modified from the DSBcapture protocol and creatively employs both the polymerizing and exonuclease activities of a specific DNA Polymerase I (DNApolI). DNApolI resects the 3′ overhangs and fills in 5′ overhangs to create blunted ends compatible with the remainder of the DSBcapture workflow. Upon sequencing alignment, the exact break location can be deduced based on the shift of the CNCC value of the reads. The extent and direction of the shift corresponds to the length and chemistry of the overhang on the endogenous DSBs. Traditionally, DSB detection methods have used non-specific end resection and thus, lost some level of resolution. CNCC-seq corrects this and can be used to map DSB end structure *a priori*, lending itself to the investigation of DNA damage repair fates in the presence of genotoxic drugs or of novel motif-associated DNA end structures.

### sBLISS

The most recent advancement in the BLESS family of genome-wide DSB mapping methodologies is sBLISS (in-suspension breaks labeling *in situ* and sequencing) (Bouwman 2020). With sBLISS, Bouwman and colleagues developed a more manageable and scalable protocol suitable for cell suspensions. Compared to previous DSB mapping protocols, sBLISS differs primarily in cell harvesting and in sBLISS template preparation. Cells in culture, tissue or tumor biopsies, and isolated nuclei can be harvested for sBLISS. The cells are then cross-linked with paraformaldehyde, lysed, and nuclei are isolated and permeabilized. After DSB ends are blunted *in situ*, the sBLISS adaptors, composed of a sample barcode, UMI, TruSeq Small RNA “RNA 5 (RA5) Illumina adaptor,” and a T7 promoter, are then ligated to the blunted DSBs. After the DNA is extracted, purified, and sonicated, the DSB ends are amplified by the T7 RNA polymerase. The RA3 Illumina adaptor is then ligated to the amplified RNA and the RNA is reverse transcribed. The molecules are then amplified in a more efficient PCR step and sequenced. The major advantages of the sBLISS protocol are its flexibility, scalability and practicality. sBLISS is flexible in that it may be applied to any cell type or nuclei that can be suspended or isolated. The workflow is also adaptable to a high-throughput setup and supports the integration of robotics. The lack of agarose plugs reduces the input size requirement. sBLISS can be completed within 2 weeks and allows for +4C storage of fixed cells. Thus, samples can be readily shipped between collaborators and does not require careful handling of glass coverslips, making it more practical protocol compared to BLISS. Like other methods, a limitation of sBLISS is that DSBs bound by repair proteins that protect DNA ends will not be detected, while artifact may arise from DSBs that are introduced during sample processing. Taken together, the recent development of sBLISS overcomes several limitations of previous methods by increasing flexibility, scalability and practicality.

## Comparison of DSB Mapping Techniques

The rapid development and refinement of genome-wide, nucleotide resolution DNA DSB mapping techniques has given rise to many, sometimes quite similar, methods. As often is the case, the experimental question and process will be the best guide to select the appropriate technique. To aid in this selection process Table 1 compares these techniques and lists some distinguishing advantages and disadvantages of each method. Some important considerations when designing an experiment with these methods are sample type and abundance, predicted signal strength (i.e., signal to noise ratio), cell and nuclei size, need for quantification of DSBs per cell and desired throughput. For example, when designing an experiment with a limited number of primary cells, one may consider BLISS or sBLISS which are more suitable for low input samples because they do not require agarose embedding. However, the agarose embedding step such as in the END-seq method may help to reduce background noise to enable detection of less abundant DSBs. In addition, when precise quantification of DSBs per cell is needed, the “spike-in” method of qDSB-seq would be more suitable. As another example, if knowing the exact end structure of the DNA DSB is critical to the biological question, one would likely consider CNCC-seq which is capable of mapping DNA DSB end structures at nucleotide resolution throughout the genome—a unique advantage since all other methods include some form of end processing. Lastly, it is important to note weaknesses of current methods: in order to minimize contribution from non-endogenous breaks formed during sample processing, all current methods employ the *in situ* steps of tagging endogenous DSBs. However, it is not clear what fraction of endogenous DSBs are not accessible, (e.g., obscured by interactions with DNA binding proteins) under these conditions. Another weakness is that several methods require DSB end processing steps that can shift the annotated location from the original break site (Figure 1). Moreover, endogenous processing of the break site may similarly cause a shift from its original location. Overall, the current catalog of DSB mapping techniques requires careful consideration of relative advantages and limitations so that the most appropriate method can be selected (Table 1).

**Table 1:** Comparison of genome-wide, nucleotide resolution methods for mapping DNA damage.

	**Technique (year)**	**Input Cells (type & number)**	**Agarose/ Fixation**	**DNA Frag. Method**	**Seq. Method**	**Advantages**	**Disadvantages**	**References**
DSB	BLESS (2013)	U2OS, HeLa, mouse lymphocytes ≥ 1 × 10^6^	Fixation	HaeIII digestion	Sanger, Roche 454, Illumina	First technique to map DSBs genome-wide at nucleotide resolution	High background relative to subsequent methods; low-speed centrifugation not suitable for small nuclei	Crosetto et al., 2013
	END-seq (2016)	Abelson-transformed pre-B cells ≥ 1.5 × 10^7^	Agarose	Sonication	Illumina	Improved sensitivity (number of possible sites detected) and accuracy (distance from predicted site) compared to BLESS	Agarose embedding may help to decrease background, but increases required number of input cells	Canela et al., 2016
	DSB-Capture (2016)	U2OS (AID-DIvA), NHEK, HeLA ≥ 1 × 10^6^	Fixation	Sonication	Illumina	Improved sensitivity (number of possible sites detected) compared to BLESS	Low-speed centrifugation not suitable for small nuclei	Lensing et al., 2016
	BLISS (2017)	KBM7, U2OS, HEK 293, mouse ESCs, mouse liver tissue sections ≥ 1 × 10^5^	Fixation	Sonication	Illumina	Suitable for low-input samples and tissue sections; more quantitative than BLESS due to unique molecular identifiers (UMIs)	Glass coverslips can cause technical challenges due to breakage or loss of cells which increases variability; not scalable for high throughput experimentation	Yan et al., 2017
	i-BLESS (2018)	*S. cerevisiae* ≥ 2.5 × 10^9^	Both	Sonication	Illumina	Agarose beads more efficient than agarose plugs; optimized fixation for low background and sample storage	Requires high-input samples; only performed in yeast	Biernacka et al., 2018
	qDSB-seq (2019)	*S. cerevisiae*, U2OS (DIvA)	Both^*^	Sonication	Illumina	Quantifies DSB frequency per cell in addition to mapping locations; integrates well with other techniques	More computationally and experimentally complex than other methods	Zhu et al., 2019
	CNCC-seq (2020)	human lymphocyte cell line (GM13069) ≥ 1 × 10^6^	Fixation	Sonication	Illumina	Only method that allows *a priori* elucidation of DSB end structure, including TOP2 cleavage sites and resection progression	Reliance on CNCC values limits cross comparison between methods and requires specialized computational analysis	Szlachta et al., 2020
	sBLISS (2020)	K562, TK6, primary CD34+ progenitors, MCF10A-AsiSI, Caco-2, mouse enterocyte tissue ≥ 1 × 10^6^	Fixation	Sonication	Illumina	Suitable for cell suspensions; scalable for high throughput; improved library preparation compared to BLESS or BLISS	Compatible with, but not as quantitative as qDSB-seq; centrifugation step may need to be optimized for cells of different sizes	Gothe et al., 2019; Bouwman et al., 2020
SSB	SSiNGLe (2019)	K562, mouse N2a, HeLa, human PBMCs ≥ 1 × 10^6^	Fixation	MNase digestion	Helicos SMS, Illumina	First technique to simultaneously map SSBs and DSBs genome-wide at nucleotide resolution	Only detects endogenous SSBs with a 3′OH group	Cao et al., 2019
	GLOE-seq (2020)	*S. cerevisiae*, HCT116 ≥7 × 10^5^	Agarose	Sonication	Illumina	Maps Okazaki fragments with significantly fewer cells compared to OK-seq (Petryk et al., 2016)	Only detects endogenous SSBs with a 3′OH group; processing in agarose plugs may lead to loss of <1,000 bp fragments	Sriramachandran et al., 2020
	Nick-seq (2020)	*E. coli, S. enterica* 1 μg of DNA (input number not reported)	Neither	NciI; HindIII and XhoI; or SalI, XbaI and NdeI digestion	Illumina	Increased accuracy due to combination of information from two experiments	May not be applicable to more complex breakomes (e.g., eukaryotic cells); absence of fixation or agarose embedding may increase background	Cao et al., 2020

* *Followed similar DNA purification protocols to the BLESS and i-BLESS methods using fixation and agarose beads, respectively, but not concomitantly*.

## Low Resolution SSB Detection

### SSB-seq

In 2014, Baranello et al. developed a method for mapping SSBs genome-wide they called SSB-seq (Baranello et al., 2014). In this method, DNA is isolated and SSBs are labeled by nick translation with digoxigenin-modified nucleotides using DNA polymerase I. After shearing, SSB fragments are captured with an anti-digoxigenin antibody and located by sequencing on the Illumina platform. Performing SSB-seq on a colon cancer cell line (HCT116), Baranello and colleagues observed enrichment of DNA breaks at transcription start sites (TSS). Further, the level of damage positively correlated with gene expression; that is, highly expressed genes tended to have more breaks at their TSS. This observation supports a relatively recent theory of DNA damage-mediated transcriptional activation [for review consider (Vitelli et al., 2017)]. SSB-seq is more precise than ChIP-seq of DNA damage-associated proteins like γH2AX, which can span thousands of bases upstream and downstream from the lesion. However, SSB-seq cannot resolve DNA breaks at nucleotide level. Also, SSB-seq requires purified DNA as input that might increase background of non-endogenous breaks. Parenthetically, Baranello et al. also developed and performed these experiments with DSB-seq, a non-nucleotide resolution method to map DSBs genome-wide as described above.

## Nucleotide Resolution SSB Detection

### SSiNGLe

**Figure 2:**
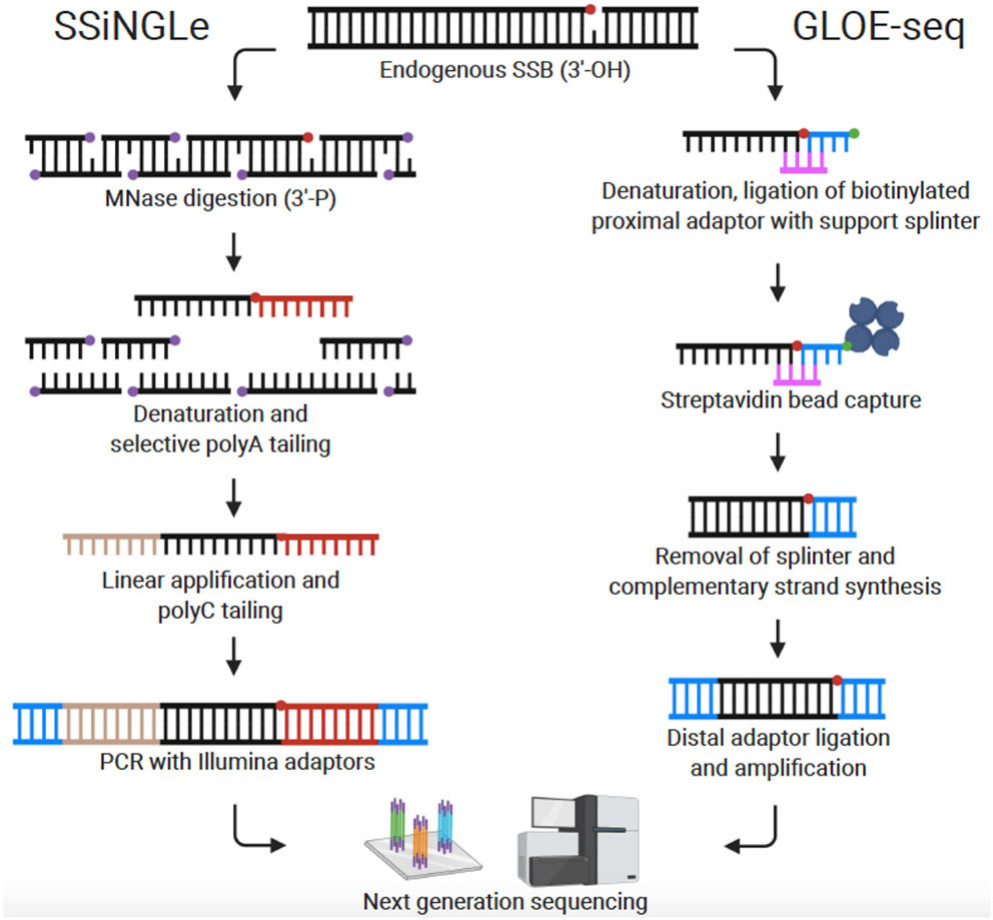
Comparison of genome-wide, nucleotide-resolution SSB mapping techniques. Created with BioRender.com.

Recently, Cao et al. published the first genome-wide, nucleotide resolution SSB mapping technique, termed SSiNGLe (single-strand break mapping at nucleotide genome level) (Cao et al., 2019; Gao et al., 2020). SSiNGLe relies on labeling DNA breaks according to the unique chemistry associated with different types of DNA damage. Native SSBs have an exposed 3′-OH group at the site of the break. To avoid background from non-endogenous breaks caused by mechanical shearing, DNA within fixed nuclei is fragmented by Micrococcal nuclease (MNase) which produces 3′-phosphate termini. Thus, native SSBs can be selectively labeled with TdT which attaches a polyA tail onto 3′-OH groups, but not 3′-phosphate groups. After selective tailing of native SSBs, the DNA is sequenced on the Illumina platform and the locations of SSBs across the genome determined with nucleotide resolution based on the location of polyA tails (Figure 2). Of note, DSBs can also be located by detection of simultaneous breaks on both DNA strands. Thus, all DNA breaks (both DSBs and SSBs) across the genome can be compiled to reveal a comprehensive DNA breakome at nucleotide resolution.

When describing SSiNGLe, the authors made several important observations: (1) consistent with the results of Baranello et al. using SSB-seq, H. Cao et al. observed enrichment of SSBs within promoters using SSiNGLe. Furthermore, H. Cao et al. observed enrichment of SSBs in other regulatory regions such as enhancers and insulators. (2) H. Cao et al. found that DNA breakage patterns in primary blood mononuclear cells (PBMCs) correlate with biological age—demonstrating the age-sensitivity of the SSiNGLe breakome and its potential to infer biological states. (3) In K562 cells (a non-neuronal cell line), H. Cao et al. discovered enrichment of SSBs in exons of genes associated with neuronal functions; the full implications of this result have yet to be understood. (4) Highlighting the importance of using high-resolution techniques, H. Cao et al. observed increased SSBs in relatively short genomic regions (e.g., exons) as well as increased breaks on template vs. non-template strands. Importantly, both of these results would not be possible to resolve with lower resolution methods. (5) H. Cao et al. found that the locations of SSBs correlated with sequence variance (i.e., mutations). This last result is particularly intriguing because of its implications for inherited and somatic genetic variation. Thus, one direct application for SSiNGLe is to examine potential mutations caused by genotoxic chemotherapy agents. Another application of SSiNGLe is to study neurodegeneration. To our knowledge, despite finding enrichment of SSBs in neuronal genes, SSiNGLe has only been performed with cancer cell lines or PBMCs.

### GLOE-seq

Sriramachandran et al. also published a method for mapping SSBs at nucleotide resolution which they termed GLOE-seq (Sriramachandran et al., 2020). The basis of the technique is comparable to SSiNGLe in that SSBs are identified in eukaryotic cells based on their 3′-OH moiety. However, instead of polyA tailing, biotinylated adaptors are ligated to the 3′-OH end of SSBs in isolated and denatured DNA. DNA is then fragmented, and the adaptors captured with streptavidin beads. In preparation for Illumina sequencing, complementary strands are synthesized, and distal adaptors ligated (Figure 2). In contrast to the results reported with SSB-seq and SSiNGLe (Baranello et al., 2014; Cao et al., 2019), using GLOE-seq, Sriramachandran et al. found that SSBs were enriched at transcription termination sites (TTS) and underrepresented at transcription start sites (TSS). Reasons for this discrepancy are still speculative, but the disparity could stem from differences in methods or cell types utilized. A side-by-side comparison of SSiNGLe and GLOE-seq will be needed to resolve inconsistent findings. A notable procedural difference between SSiNGLe and GLOE-seq is the use of agarose plugs to reduce artificial breaks, similar to methods discussed above for DSB detection. Unlike SSiNGLe, where cells are not embedded in agarose, with GLOE-seq, the initial steps (up to cell lysis) are performed on cells embedded in an agarose plug. The necessity and effectiveness of agarose plugs for SSB mapping has not been determined; again, highlighting the need for future inter-protocol comparison. Taken together, both GLOE-seq and SSiNGLe are key SSB mapping methods. A critical next step for SSB mapping is to extend this technique to more human cell lines and disease states including cancer and neurodegeneration.

### Nick-seq

Around the same time that GLOE-seq was published, another novel quantitative method was developed by B. Cao et al. to perform single nucleotide resolution genome-wide mapping of a wide variety of DNA modifications in *E. coli* (Cao et al., 2020). This method is unique in a number of ways, the most notable being their simultaneous use of complementary nick translation (NT) and TdT strategies to capture free 3**′**-OH ends. After treating cells and purifying genomic DNA, samples are split in half and processed using either the NT or TdT method. Requiring reads from both strategies to align increases the specificity of accurately mapped reads from around 95% (TdT alone) to over 97% and reduces false positives. The authors also curated a list of DNA modification-dependent restriction endonucleases (MRE) and other well-characterized DNA damage repair enzymes for use with Nick-seq to convert specific DNA modifications into backbone nicks compatible with the Nick-seq workflow. They validated the versatility of Nick-seq by converting phosphorothioate DNA modifications and AP sites to 3′-OH nicks using the iodine-phosphatase method and EndoIV, respectively. The high precision of detection is a unique strength of the Nick-seq method. However, the requirement for breaks to be detected by both approaches is also a potential limitation since only high-frequency breaks could theoretically be detected. Furthermore, signal from nearby breaks would overlap and thus likely present an additional analytical problem. Finally, just like SSB-seq, Nick-seq requires purified DNA as input which might create background of non-endogenous breaks. Combined together, these issues may limit application of Nick-seq in the analysis of breakome from complex genomes. However, despite these limitations, there is great potential for blending components of each of these SSB detection methods to test a range of hypotheses related to the SSB breakome (Table 1).

## Current and Future Applications

Here we have reviewed several recently developed techniques capable of mapping DNA breakage. Many of these techniques have been spurred, at least in part, by the recent CRISPR revolution and the need for methods to detect off-target DNA damage or modification, although other techniques have been developed specifically for that purpose (Cameron et al., 2017; Tsai et al., 2017; Kim and Kim, 2018). In the same vein, others have begun to use these methods to evaluate the genotoxic effects of various therapies (Cao et al., 2019). However, despite the robust link between DNA damage and neurodegeneration (Madabhushi et al., 2014; Copped and Migliore, 2015; Wei et al., 2016; Massey and Jones, 2018; Penndorf et al., 2018; Farmer et al., 2020; Lin et al., 2020), few of these techniques have been used to map DSBs or SSBs related to any neurological disease. Recently, Break-seq was used to map DSBs in cells derived from fragile X syndrome patients (Chakraborty et al., 2020). In this study, Chakraborty et al. observed increased DSBs in patient cells, particularly when under replication stress. Although Break-seq is not a nucleotide resolution technique (median resolution is ~200 bp), the authors compared the locations of DSBs to sequences predicted to form DNA:RNA hybrid structures called R-loops and discovered increased overlap between DSBs and sequences prone to R-loop formation in fragile X cells. This corroborates a growing body of evidence implicating R-loops and their mediators as key players in multiple neurodegenerative diseases [for review consider (Crossley et al., 2019; Perego et al., 2019)]. Future studies following a similar framework to study DNA damage in neurodegenerative disorders will be vital to understanding how genome instability contributes to the pathobiology of these diseases.

Multiple compelling reasons exist for expanding the use of these genome-wide, nucleotide resolution DNA damage mapping techniques to study neurodegeneration: (1) understanding where DNA damage accumulates during disease and aging could uncover underlying, and potentially unifying, disease mechanisms; (2) understanding the link between DNA damage and sequence variance could help prevent accumulation of deleterious mutations; (3) determining genomic locations susceptible to incurring DNA damage during disease could help define treatments to combat disease progression; and (4) unique disease-specific DNA damage patterns could be used as a potential peripheral biomarker for the presence and progression of disease. Furthermore, the recent advent of induced pluripotent stem cell (iPSC) technologies provides unprecedented access to unlimited, modifiable, disease-relevant, human neuronal systems including iPSC-derived neurons and brain organoids. Thus, profiling disease-specific DNA damage patterns with these systems may have profound implications for neurodegenerative disease research.

## Concluding Remarks

An abundance of evidence implicates DNA-damage induced genome instability as an important driver of human diseases, especially cancer and neurodegeneration. Valiant research efforts in recent years have inaugurated the “breakome” era—where new comprehensive technologies allow for the study of DNA damage and repair genome-wide at nucleotide resolution. The application of these new technologies will certainly lead to an increased understanding of how impaired DDR and repair contributes to disease.

## Author Contributions

MJR, MR, and NR wrote and prepared the original draft with the guidance of ZZ. MJR created the figures. ZZ made significant edits with expert input and revisions by CW and PK. All authors read and approved the final version.

## Conflict of Interest

The authors declare that the research was conducted in the absence of any commercial or financial relationships that could be construed as a potential conflict of interest.
